# Topic modeling of maintenance logs for linac failure modes and trends identification

**DOI:** 10.1002/acm2.13477

**Published:** 2021-11-29

**Authors:** Hongguang Yun, Marco Carlone, Zheng Liu

**Affiliations:** ^1^ Faculty of Applied Science University of British Columbia Kelowna British Columbia Canada; ^2^ Department of Medical Physics BC Cancer Kelowna Kelowna British Columbia Canada; ^3^ Department of Physics University of British Columbia Kelowna British Columbia Canada

**Keywords:** failure modes, latent Dirichlet allocation, linac, natural language processing, topic modeling

## Abstract

**Purpose:**

Medical linear accelerators (linacs) can fail in a multitude of different manners due to complex structures. An unclear identification of failure modes occurring constantly is a major obstacle to maintenance arrangements, thereby may increasing downtime. This study aims to use natural language processing techniques to deal with the unformatted maintenance logs to identify the linac failure modes and trends over time.

**Materials and methods:**

The data used in our study are unformatted narrative maintenance logs recording linac conditions and repair actions. The latent Dirichlet allocation‐based topic modeling method was used to identify topics and keywords regarding the failure modes. The temporal analysis method was applied to examine the variation of failure modes over 20 years.

**Results:**

Based on the output of the topic modeling, 28 topics and keywords with frequency ranking were generated automatically. The latent failure modes in topics were identified and classified into six main subsystems of linacs. Furthermore, by using the temporal analysis method, the trends of all failure modes over 20 years were illustrated. Half of the topics demonstrated variations with three different patterns, namely periodic, increasing, and decreasing.

**Conclusions:**

The results of our study validated the effectiveness of using the topic modeling method to automatically analyze narrative maintenance logs. With domain knowledge, failure modes of linacs can be identified and categorized quantitatively.

## 1 INTRODUCTION

Medical linear accelerators (linacs) are the most important equipment in radiotherapy. At their core, these are particle accelerators reconfigured as medical devices. The mode of operation of the linacs uses similar physical principles as high‐energy accelerators for particle physics research. However, the medical devices operate in hospital environments that pose additional challenges for operations due to the different physical environments and availability of technical support. Operating medical linacs requires skilled personnel to repair, adjust, and otherwise maintain the proper operation of the devices. Further, medical linacs have many subsystems that all must operate faithfully for the device to function correctly. Given their complexity, linacs can fail in a multitude of different manners.

It is desirable to understand the nature of medical linac failures for several reasons. First, components used in medical linacs are costly, and improved knowledge of components that fail more often can be of help in projecting service and maintenance costs for medical linacs. Second, the training of the qualified technical staff able to maintain these devices can be simplified with better knowledge of failure modes since emphasis can be placed on areas that fail more frequently. Third, a better understanding of failure modes can help medical linac operators in stocking components that are more likely to be needed in maintenance, which can help reduce repair times.

There have been relatively few studies of failure modes for linacs. Wroe and colleagues^1^ studied downtime and failure modes for radiotherapy equipment in lower income and developed countries. Sheehy and colleagues^2^ performed a reliability analysis of radiotherapy equipment in lower income countries. Both studies commented on the difficulty in obtaining sufficient quality data and statistics to conduct their analysis. The first study relied on the manual review of linac maintenance records, in both electronic and paper form to estimate failure modes and times between failure. The second study improved in the first by building failure mode models using its results.

Modeling of linac failure modes would be greatly simplified and improved with high quality and consistent input data for the model development. However, medical linac maintenance data are often kept in generic equipment maintenance databases whose primary purpose is to keep records of maintenance, but not to classify and analyze the failure modes. The core information in maintenance logs is recorded in narratives by maintenance personnel. In general, these logs depict the repair procedures and maintenance results, making it useful for analysts to evaluate the linac performance and identify failures. But these logs have the characteristics of colloquial, unformatted, noisy, and may contain spelling and grammatical errors, which is not suitable for general analytical tools. In particular, it is time‐consuming to analyze this type of data by humans when the scale of data is large.

This research is triggered by the natural language processing (NLP) application in the transportation domain, where researchers applied topic modeling (TM), a type of unsupervised learning algorithm, into categorizing safety reports for reducing incidents’ events.^3^, ^4^ The NLP technology is established with a suite of methods capable of interpreting, evaluating, and generating narratives in human language. There has been a slightly increased amount of literature on medical records analysis during the COVID‐19 pandemic. Shah et al.^5^ carried out a number of investigations into the patient online reviews in physician rating websites to examine trends of patient concerns due to the COVID‐19. The coherence‐based TM method was applied to generate topics and corresponding keywords and experiment results showed that policymakers can benefit from the topic analysis to deal with the COVID‐19 crisis efficiently. Kaveh‐Yazdy and Zarifzadeh^6^ investigated the top‐ranked people concerns to the COVID‐19 in Iran. Based on the output of the TM model, researchers summarized the major concerns are PCR lab and test, policy on the education system, and personal protection actions such as wash hands and wear masks. In our study, the emphasis was placed on the maintenance work of linacs, where the TM method was applied to analyze the massive and unformatted linac maintenance logs to identify the most frequent failure modes.

The purpose of this work is to investigate the feasibility of using TM to analyze electronic medical linac maintenance logs. The main contribution of this article is to introduce TM to analyzing the unstructured maintenance logs data to find out the most frequent failure modes of linacs during daily use. Another purpose is to demonstrate the performance of different linacs over time by examining the trends of different failure modes. With a data‐driven analysis method, it is hoped that the larger pool of current medical linac maintenance logs can be used to better understand medical linac failure modes.

## 2 MATERIALS AND METHODS

### 2.1 Linac maintenance logs

The maintenance logs used in this study were collected from several linacs of BC Cancer center, Kelowna (Canada) under the regulation designed by the Canadian Nuclear Safety Commission. The linacs were in service from April 1998 until the study date. There were nine linacs in total, four of these were replaced partway through the study period. The fifth linac was also added in 2009. These linacs were installed in five different treatment rooms, labeled A–E as shown in Table 1. The dates of service, manufacturer, and model were also listed. Of the four original linacs, two were equipped with multi‐leaf‐collimators (MLCs) and amorphous silicon‐based electronic portal imaging (EPID); the other two did not have MLCs but had fluorescence imaging‐based portal imaging which was upgraded to amorphous silicon EPIDs. The five accelerators in service from 2011 onward are modern medical linacs with MLCs, EPIDs, and kV based on‐board imaging. The dataset used in our study was from nine different linacs recorded from April 1998 to December 2019 consisting of 4323 entries in total.

**TABLE 1:** Linacs specification and service date

Treatment room	Manufacturer	Model	Starting service date	End service date
A	Elekta	SL75	April 1998	October 2008
A	Varian	Clinac iX	July 2009	September 2021
B	Elekta	SL75	April 1998	December 2009
B	Varian	Clinac iX	September 2010	January 2021
C	Elekta	SL 20	July 1998	July 2010
C	Varian	TrueBeam	March 2011	Present
D	Elekta	SL 20	July 1998	February 2011
D	Varian	TrueBeam	August 2011	Present
E	Varian	Clinac iX	November 2009	Present

The maintenance log is a collection of narrative maintenance records of linac repair and service work completed by maintenance personnel. In our study, there are two main parts in the logs, namely ‘‘Comments’’ and ‘‘Repair Description.’’ ‘‘Comments” briefly describes the linac status and breakdown occurs on the linacs. ‘‘Repair Description’’ records the maintenance procedure, repair action, and related broken component of linacs. Some metadata were also recorded, such as the date of the maintenance service. Table 2 shows two maintenance log entries sliced from the original dataset. Apart from the ‘‘Comments,’’ ‘‘Repair Description,” and ‘‘Date,’’ Keyword ‘‘TaskKey” tells the type of the maintenance service.

**TABLE 2:** Example entries of linac maintenance logs

TaskKey	Comments	Repair Description	Date
Corrective	Touchguard interlock could not be removed with iView detector in place.	Adjusted touchguard microswitches at the locking pin end for proper contact. Adjusted the alignment nuts on both sides of detector for easier locking pin insertion.	April 1998, 16
Major repairs	CARR/FOIL W29 cable repaired. Error 7F when calibrating carrousel, was also getting error 70 when exiting calibration, pointing to switch S16.	Cleaned all five switches and reseated connectors J82 and J83. Adjusted the Carr pot voltage to 5.05 V from 4.74 V. Replaced and adjusted S16 on the carrousel switch assy, no change. We could reproduce the fault by moving the gantry from Zero degrees to 350°. Lubed Carr chain with TriFlow. Replaced the PWM pcbA4, no change.	January 2020, 29

As shown in Table 2, the narratives in the logs (‘‘Comments’’ and ‘‘Repair Description’’) contain a wealth of information describing the health condition of linacs and repair action. However, to identify frequent failure modes through examining the logs, it is evident that it would be time‐consuming to extract key information from the lengthy sentences by humans, especially for the whole dataset. In such a case, by using the NLP techniques, the key information, in our case, the failure modes and related components of linacs, can be extracted automatically and quickly from the logs. Furthermore, the temporal analysis method was used by implementing the metadata ‘‘Date” to find out the trend of specific failure modes over time. As mentioned previously, the linacs have been replaced and added from different manufacturers around 2010. Thus, the temporal analysis was used to examine whether there is a difference between different linac models.

### 2.2 Topic modeling of linac maintenance logs

#### 2.2.1 Latent Dirichlet allocation model

Following the documents representation method, latent semantic indexing (LSI), Blei et al.^7^ proposed latent Dirichlet allocation (LDA) algorithm and formulated a general technique named probabilistic TM. TM is a typical unsupervised machine learning algorithm, and it doesn't require labeling the dataset but constructs a model solely on the distribution of the words in documents. TM is capable of extracting core information by distilling topics from messy documents.

LDA is the most commonly used algorithm to perform the TM in a collection of documents. LDA constructs a three‐layer architecture between documents, topics, and words by independent multinomial distributions. Each document is represented by several latent topics and each topic is governed by a multinomial distribution over words. In our application scenario, for each entry of the maintenance logs, the ‘‘Comments’’ and ‘‘Repair Description,’’ were combined as a document. All documents in the maintenance logs dataset comprise the corpus. LDA summarizes the documents with several topics by searching similar ‘‘bag” of words co‐occurring in all documents. The words with top frequency in each topic describe the core information of the topics. In our case, the top‐ranked words often point to some kinds of failure modes. Thus, the TM can help us find the most frequent failure modes by searching the keywords in the dataset. It should be noted that a single document is often represented by several topics. This is quite reasonable that one maintenance service usually handles multiple failures.

**FIGURE 1:**
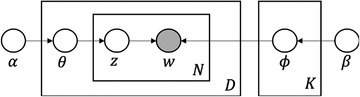
Graphical representation of latent Dirichlet allocation model

To further explain the mechanism of the LDA model. Some notations and assumptions are introduced here. A document noted as d consists of N words, where $w_{n}$ is the nth word. A topic, noted as z, is a bag of semantic words and can be expressed by the z:{w,p(w|z,β)}. The topic is indexed by k which goes from 1 to K. The process of building an LDA model was displayed in a ‘‘plate‐like’’ graphical model as shown in Figure 1.^7^ In the graphical model, nodes represent variables and the arrows represent the variable dependency. Plates denote repeated sampling and the number of sampling is indicated at the bottom right corner of the box. The LDA graph model can be interpreted in the algorithm by the following steps^8^:

1. Set prior parameters: N∼Poisson(ξ),Dir(α),Dir(β).
2. For each document d, choose θ∼Dir(α).
3. For each word $w_{n}$, the topic of the word belonged denoted as $z_{n}$ is drawn from Multinomial(θ).
4. The word $w_{n}$ itself is a variable drawn from another distribution $\mathrm{Multinomial}(w_{d}|z_{n},\phi )$.

Here, αand β are two independent symmetric Dirichlet priors; θ and ϕ are the document‐topic distribution and topic‐word distribution drawn from Dir(α)and Dir(β), respectively.

The joint distribution of z,θ, and w is given by

(1) $$p\left(\theta ,z,w\left|\alpha ,\beta \right.\right)=p\left(\theta \left|\alpha \right.\right)\prod_{n=1}^{N}p\left(z_{n}\left|\theta \right.\right)p\left(w_{n}\left|z_{n},\beta \right.\right)$$

By applying the Bayes’ theorem, the topic mixture θ can be obtained by computing the posterior distribution:

(2) $$p\left(\theta ,z\left|w,\alpha ,\beta \right.\right)=\frac{p\left(\theta ,z,w\left|\alpha ,\beta \right.\right)}{p\left(w\left|\alpha ,\beta \right.\right)}.$$

#### 2.2.2 Metrics for topic modeling parameter fine‐tune

The number of topics K is the most important parameter need to be determined when training an LDA model. A too‐small K will concentrate too much information on a single topic, making it difficult to identify the specific failure mode and map it to the responsible components. Likewise, a too big Kwill make the information too scattered, leading to some meaningless topics. However, the TM is an unsupervised method, there is no ground truth to provide reference for selecting the optimal number of topics. Thus, some metrics are proposed to help to address this issue by evaluating the topic model.^9^, ^10^, ^11^, ^12^, ^13^

Evaluation metrics can provide a good reference for finding the optimalK. Kuhn^3^ used a pair of trade‐off metrics, namely coherence and exclusivity, and selected the outlier as the optimal K. Wang et al.^14^ selected Jensen–Shannon divergence and perplexity to find the optimal metrics. Another more direct way is to check the result of the models and discern whether it is reasonable.^15^ Tanguy et al.^16^ chose 50 as the optimal K by a subject matter expert in the application of analyzing the aviation safety reports.

The divergence metric proposed by Arun aims to find the optimal K by computing the Kullback–Leibler divergence of topic–word distribution over singular values and is defined as^11^:

(3) $$\begin{array}{ccc} \mathrm{Divergence}\left(M1,M2\right) & = & \mathrm{KL}\left(C_{M1}∥C_{M2}\right)\\ & & +\;\mathrm{KL}\left(C_{M2}∥C_{M1}\right), \end{array}$$

where $C_{M1}$ is the distribution of singular values of the topic–word matrix M1, and $C_{M2}$ is the distribution of the normalized document–topic matrix M2.

Several experiments showed the optimal K can be reached by minimizing the proposed metric. Another commonly used metric is perplexity, which is an indicator of the uncertainty of a model in predicting the topic of the held‐out data. The perplexity is “equivalent to the inverse of the geometric mean per‐word likelihood” in mathematics:

(4) $$\mathrm{Perplexity}=\exp\left\{-\frac{\sum_{d=1}^{M}\log p\left(w_{d}\right)}{\sum_{d=1}^{M}N_{d}}\right\},$$

where a model with lower perplexity tends to give a more reasonable prediction. A trial and error process would be needed if the perplexity and divergence become a pair of trade‐off metrics for a specific problem.

The above two metrics are statistical evaluation methods. However, the perplexity is shown insufficient to determine the optimal K in some real applications. Thus, another metric called coherence was proposed to evaluate the interpretability of the model.^17^ The coherence is established on the assumption that a topic is easier to interpret when its top‐ranked words co‐occurred more frequently in the documents of the corpus. For example, a topic with the top words ‘‘MLC’’ and ‘‘leaf’’ is easy to interpret as these two words co‐occur many times in different log pieces. The coherence is defined as follows^17^:

(5) $$\mathrm{Coherence}=\sum_{m=2}^{M}\sum_{l=1}^{m}\log\frac{D\left(w_{m}^{k},w_{l}^{k}\right)+1}{D\left(w_{l}^{k}\right)},$$

where $W^{k}=\left(w_{1}^{k},\text{…},w_{M}^{k}\right)$ is the list of M top words in topic k,D(w) is the number of service records that contain the word w, and $D(w,w^{\prime })$ is the number of service records in which both words w and $w^{\prime }$ occur at least once. Generally speaking, the coherence will decrease as the number of topics in a model increases and the model with a higher coherence score is more interpretable.

In this article, perplexity, divergence, and coherence were used to determine a narrow range of optimal K. The final optimal number of topics is selected by three subject experts by checking the model interpretability.

### 2.3 Temporal analysis of linac failure modes

The most frequent failure modes of linacs may change as the service time increases. To examine whether there is performance degradation of specific subsystems in linacs, the occurrences of different failure modes in each year should be analyzed. As there is a replacement of linacs from different manufacturers around 2010, the analysis over time can be used to compare the performance of linacs from different manufacturers. The temporal analysis method was used to investigate the trends of different failure modes over years.^18^ As the metadata ‘‘Date’’ is available, each entry in the maintenance log was assigned to a specific date, and each entry was modeled as a topic cluster about failure modes through the LDA model. Thus, the proportion of any failure modes over years can be obtained by calculating the probability $p(z_{j}|y)$, of a given year $z_{j}$ ^9^:

(6) $$p\left(z_{j}|y\right)=\frac{1}{n_{y}}\sum_{d:t_{d}=y}\theta_{j},$$

where $\theta_{j}$ is the document–topic distribution and $t_{d}=y$ is the year that the document was recorded.

The NLP is able to analyze phrases or combinations of words by building a language model using *N*‐grams. An *N*‐gram is a sequence of *N* words. An *N*‐gram language model predicts the probability of a given *N*‐gram within any sequence of words in the language. The most commonly used are bigram and trigram models. In our work, we built two topic models using bigram and trigram, respectively.^19^ In our experiment, the bigram‐based model outperformed the trigram‐based model. The results in the article were based on the bigram model. It is worth noting that the maintenance logs are noisy and often includes meaningless and incorrect information such as spelling mistakes. Several text pre‐processing techniques were employed before building an LDA model, including the following:

1. *Tokenization*: Token is the basic element in a topic model. This process breaks up the sentence into an individual token for the following processing and analysis.
2. *Words cleaning*: Remove punctuation characters, numbers, and stop words that highly frequent occurring in most topics while contributing little to the topic building, such as preposition words and etc. In addition, any words that occurred in the whole dataset less than three times were removed.
3. *Lemmatization*: Lemmatization aims to return the base or dictionary form of words so that they can be treated as a single element via TM. Another more aggressive technique called stemming is tested ineffective because it may combine distinct tokens as one and convert jargon in the linac field to some other words.
4. *Lowercase conversion*: Convert words to lowercase.

## 3 EXPERIMENTS AND RESULTS

There are three parts in this section. First, the process of selecting the optimal K using the metrics mentioned in Section 2 was demonstrated. Then, the method of interpreting the topic contents produced by the LDA model was demonstrated and the most frequent failure modes of linacs were summarized. Finally, the temporal analysis method was used to identify the trends of some failure modes.

### 3.1 Experiments on LDA model of maintenance logs

#### 3.1.1 Topic number selection

**FIGURE 2:**
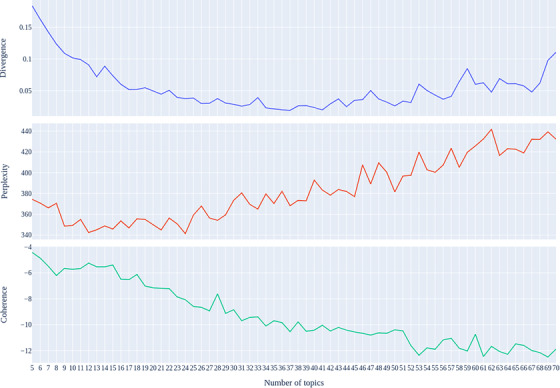
Metrics used to select the optimal number of topic

The number of topics K is the most significant parameter in building a good LDA model. To find the optimal K, three metrics mentioned in Section 2 were used to evaluate LDA models with different K. Figure 2 illustrates the trends of divergence metric, perplexity, and coherence as the number of topics increases from 5 to 70.

The value of divergence reaches a lower level, which indicates a possible good LDA model when the topic numbers were set from 25 to 50. However, the perplexity increases as the topic numbers exceed 25. Since the model with a smaller perplexity has a better prediction power for new data, these two metrics become a pair of trade‐off metrics for this problem. Thus, the third metric coherence is used to determine the final interval of the optimal K. Nevertheless, the range of optimal K can be initially determined from 25 to 35 according to the above two indicators. Furthermore, from the figure, we can find the coherence value of the model with 28 topics standing out compared to others in the initial range from 25 to 35. With the help from the subject matter expert interpretation of the models with K range from 25 to 30, 28 was selected as the optimal K in the following analysis. Finally, the following analysis is based on the model with 28 topics.

#### 3.1.2 Identified topics and latent failure modes

From the output of the well‐built LDA model, 28 topics were clustered with specific words selected from the linac maintenance log dataset. However, the explicit concept and topic meanings are not generated accordingly.^3^ A post‐analysis to identify the core information of the topics based on the top‐ranked words is required. With this topic interpretation procedure, the specific failure modes and related components or subsystems of linac can be identified and summarized.

The most straightforward approach to find what a topic represents would be to rank words by frequency on the topic and find the common narratives among those words. Thus, the underlying failure mode and subsystem can be found. However, it would cause a problem that some words which have a high overall frequency across the corpus would show up on many topics. These words may cover words that have a relatively lower probability while contributing a lot in interpreting topics. Therefore, another statistical metric called lift was introduced to rank the top words within topics. The lift is defined as ‘‘the probability of word occurrence conditional on topic divided by the probability of word occurrence across the corpus.’’ This metric will highlight words that have a high probability within a topic locally than those across a corpus.

To use the frequency and the lift metric more flexible, the visualization and analysis package PyLDAvis^20^ was used to sort the top‐ranked words. It introduces a parameterλ(where0≤λ≤1) to adjust the weight of frequency and lift metric given a specific word. The ‘‘relevance’’ of a word to the topic k is defined as:

(7) $$r\left(w,k|\lambda \right)=\lambda \log\left(\phi_{kw}\right)+\left(1-\lambda \right)\log\left(\frac{\phi_{kw}}{p_{w}}\right),$$

where $\phi_{kw}$ denote the probability of word w for topic k and $p_{w}$ denote the marginal probability of word w in the corpus. Given λ=1, the relevance is identical to the ranking of words in decreasing order within a topic and the relevance will equal to lift when λ=0.

**FIGURE 3:**
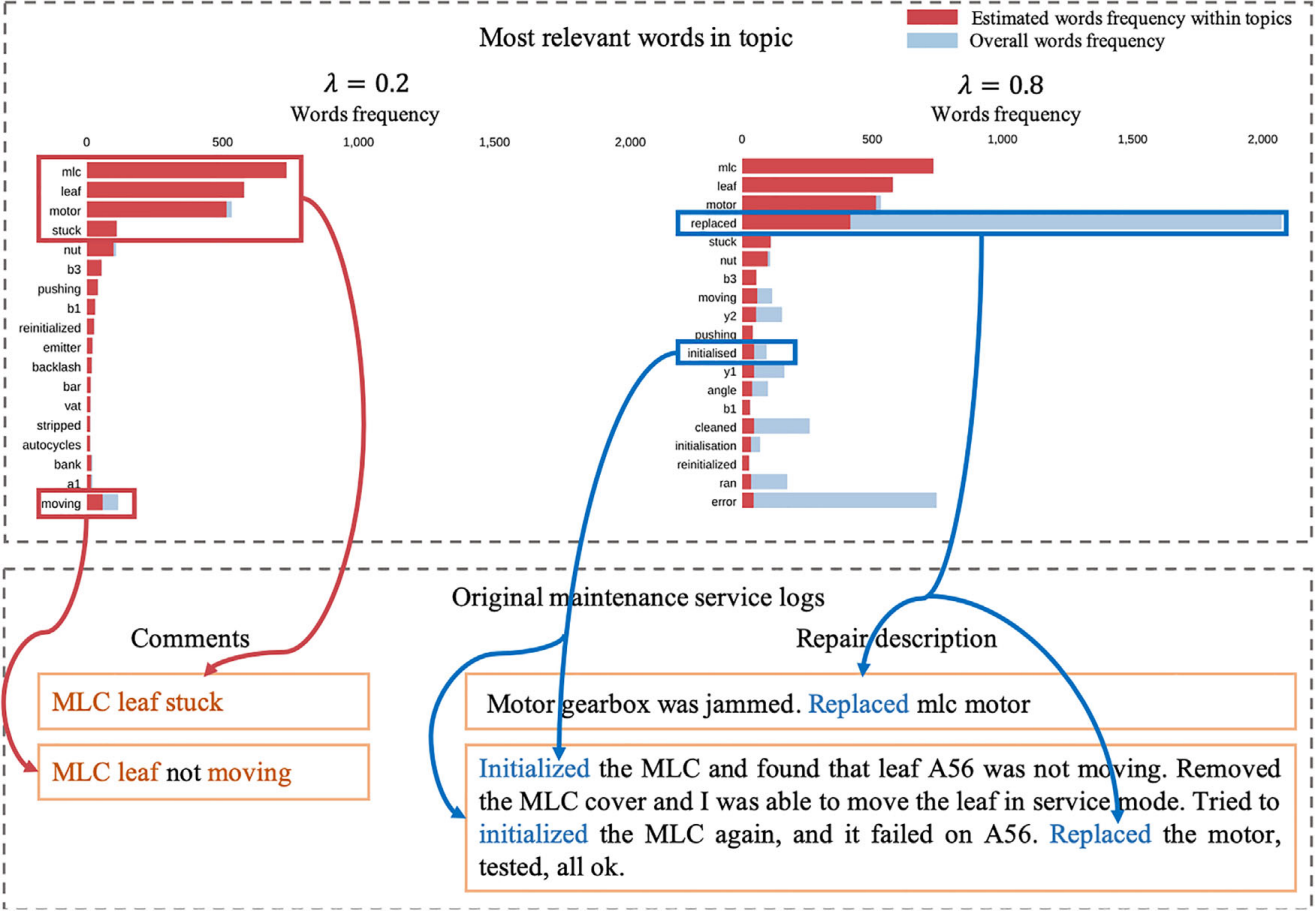
Top‐ranked words within topic 3 and corresponding failure mode

Take topic 3 as an example to demonstrate the process of determining the meaning of a specific topic. When we set λ=0.2, the words will be ranked more by their occurrence within the topic. As shown in Figure 3, the top four words are ‘‘mlc, leaf, motor, stuck,’’ which clearly point to the failure on the MLC and the failure mode is ‘‘mlc leaf stuck.” Then, we set λ=0.8, the words are ranked much more by their overall probability over the corpus. From Figure 3, the weight of the word ‘‘replaced’’ was increased and it indicates the repair action ‘‘mlc leaf motor replaced.” Therefore, it is evident that we can find the failure modes and corresponding repair action by setting the λ to 0.2 and 0.8, respectively. It should be noted that the words in a topic are not purely related to one failure mode or one component. What we are seeking is to find out the dominant words in a topic and relate these words to specific failure modes.

All 28 topics were examined by the same process to identify dominant failure modes in each topic. However, not all topics are related to specific failure modes. Some topics are generated with words describing the general maintenance work. For example, the top‐ranked words in topic 6 are ‘‘pm day routine carried maintenance,” which point to the routine maintenance records. Under this topic, the co‐occurred verbs and nouns are ‘‘cleaned, checked” and ‘‘rim, iview, and processor,” respectively. These words indicated that the main work in routine maintenance is about cleaning and lubrication. Therefore, we categorized topics with identified failure modes into related subsystems according to Wroe's paper.^1^ Furthermore, the LDA model also gave the overall proportion of words contained in each topic in the corpus. Given the assumption that topics are dominated by few top‐ranked words, the proportion of the topic reflects the frequency of the failure modes reflected in the topic. Table 3 displays the topics with top‐ranked words and identified failure modes. It is worth noting that topic 14 appeared in two subsystems as it shows up two different failure modes. A possible explanation would be these two failure modes co‐occurred many times in service. The identified topics and related failure modes are summarized below.

**TABLE 3:** Keywords within topics and identified failure modes of linacs

Subsystem	Topic (words frequency)	Keywords within topic	Identified failure modes
	Topic 2 (6.5%)	Fuse supply power tube generator relay rectifier blown outage black bridge magnetron replaced checked	Fuse blown; power outage; bridge rectifier, magnetron, modulator failure
Electrical	Topic 3 (6.4%)	MLC leaf motor stuck moving emitter reflector replaced pushing initialized cleaned	MLC motor failure
	Topic 21 (2.2%)	Reflector reference line detector lost locked verified adjusted calibration reset	MLC leaf lost reflector; reflector out of calibration
Control	Topic 4 (5.9%)	Console physics keyswitch sound timing frame board replaced check	Keyswitch replaced; buzzing sound from board
	Topic 8 (4.4%)	PCB controller carriage program fitting driver change clear tightened	Replaced controller PCB; tightened up connection
	Topic 14 (3.4%)	New Varian software version device configuration old installed upgrade completed	Software upgrade; device configuration error
	Topic 20 (2.6%)	Prescription external pair receive communication timer database clinical failure close error reboot	Reboot computer and timer; external prescription error; communication error
	Topic 25 (1.9%)	Assembly secondary carrousel radial feedback captured replaced calibrate initialize	Recalibrated carrousel; lost secondary feedback
	Topic 26 (1.7%)	OBI stand modulator charge tapping charge capacitor box assisted replaced unstuck	OBI modulator error; OBI supervisor/computer
Mechanical	Topic 5 (5.3%)	Gantry connector cable head noise display rotating patch bending monitor installed filled tightened	Gantry position error; bending system failure; tightened connector in head
	Topic 9 (4.4%)	Couch readout long tolerance rotation jaw chain final hair collimator strip encoder calibrated adjust	Gantry readout calibration; couch long calibration; adjust collimator position
	Topic 11 (3.6%)	Drive limit key bearing height thumbwheel clutch movement replaced adjusted lubricated cleaned	Carriage bearing replaced; thumbwheel replaced; couch movement failure
	Topic 12 (3.6%)	ODI lamp bulb battery green burnt emergency grid socket mod red defective pointer light replaced used reseated	ODI lamp reseated; bulb burnt; emergency battery replaced
	Topic 13 (3.4%)	Arm tray collision switch touchguard sensor actuator cover plate pin angle holder fix removed adjusted attached	Arm position error; touchguard adjustment; sensor replaced/cleaned
	Topic 15 (3.3%)	Field laser mirror alignment capital output coincidence wall vertical dim light adjusted check closed	Field lamp replaced; mirror position adjustment; coincidence check
	Topic 16 (3.1%)	Pendant tight hand latch facility information door fiber optic ribbon terminal reset replaced collect	Reset latch; pendant terminal strip; optic cable failure
	Topic 17 (1.9%)	Pot fine pro mismatch offset shaft minor gear jaw edge replaced calibrated	Jaw calibration; replaced pot
Topic 7 (4.6%)		Energy Thyratron beam URDS tuned interlock pulse voltage dequing tuned troubleshooting replaced	Thyratron failure; de‐quing and thyratron voltage low
Beam	Topic 18 (2.9%)	Gun image breaker circuit HT RF button bolt filament balance tripped pulse adjusted reset checked	Gun driver failure; adjust gun filament voltage; reset HT circuit breaker
Topic 23 (2.1%)		MV symmetry profile wedge jaws resolve steering improved adjustment repositioned	Steering adjustment
Support	Topic 10 (4.2%)	Flow fan pressure air cooled loop valve water conditioner chiller wave pumped changed shut tuned	Water contaminated; fan cooled failure; chiller pumped; conditioner fan noisy
	Topic 14 (3.4%)	Hose pump water leak tank pump calibrated gas pop up	Hose water leak; water tank calibrated; pop‐up gas
Dosimetry topic 24 (2.1%)		Ion chamber channel reading aria responding treatment dose dosimetry replaced check set calibrated	Replaced ion chamber; dose board failure; dosimetry calibrated

### 3.2 Experiments on temporal analysis of topic trends

The metadata “date” in service logs is used here to develop estimates of the topic distribution over years. The distribution of topics was obtained by calculating the probability $p(z_{j}|y)$, of a given year $z_{j}$. By examining the topic trend over years, we can find out the variation of specific failure modes.

The distribution of 28 topics can be divided into three patterns, namely constant, periodic, and quasi‐monotonic. About half of the 28 topics with constant patterns do not show large variation over years. Their probability fluctuates slightly around a value. In other words, most failure modes occur constantly. However, what we are interested in are those topics with a periodic pattern or a quasi‐monotonic pattern. Examining the causes resulting in the change of the failure modes can help us better understand the performance of the linac and arrange maintenance service. According to our results, two topics showed a periodic pattern, and eight topics demonstrated a quasi‐monotonic pattern, four topics with an increasing trend, and four with a decreasing trend.

#### 3.2.1 Periodic pattern

**FIGURE 4:**
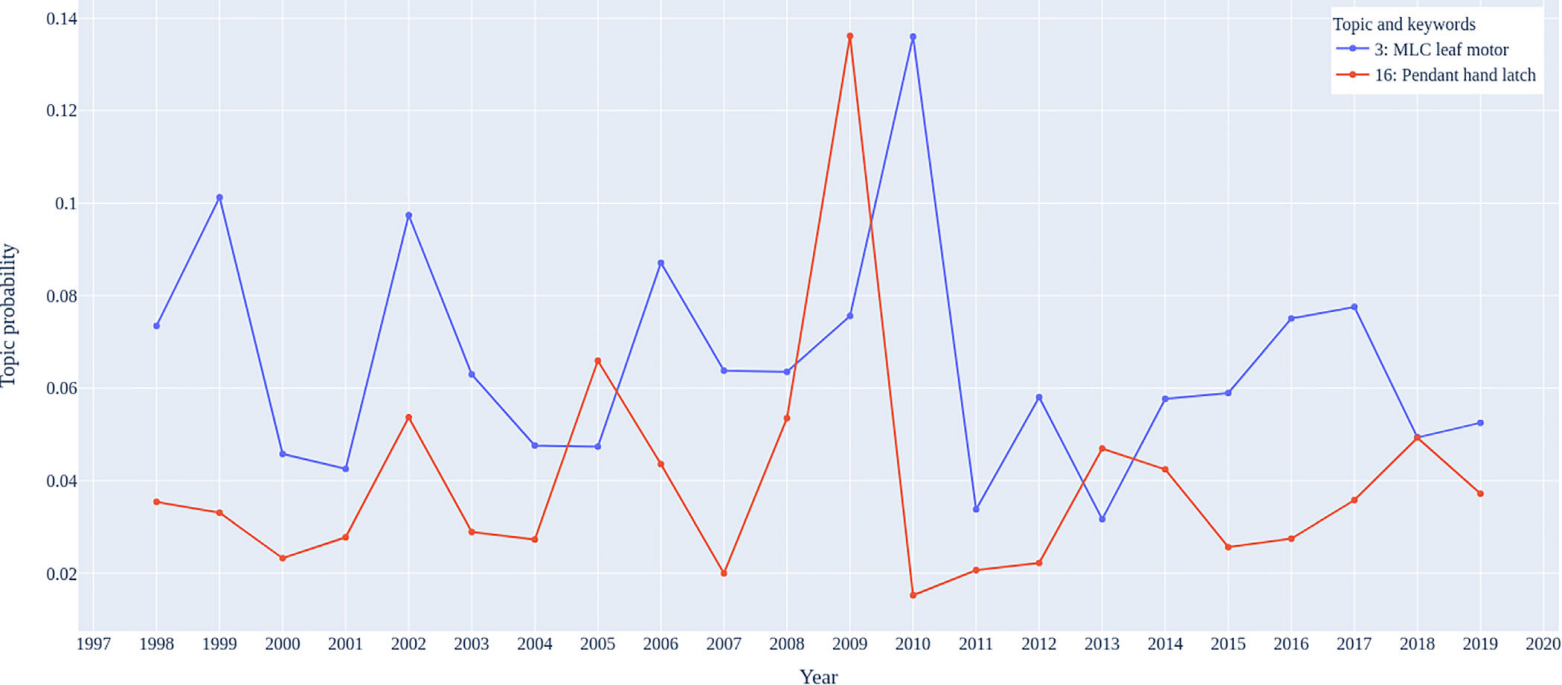
Trends of topics with periodic pattern over years

As shown in Figure 4, topic 3 and topic 16 exhibit a periodic characteristic over the last 20 years with an almost synchronous trend. Keywords within topic 3 are about MLCs’ component and point out the most frequent failure mode as MLC leaf stuck. Topic 16 is interpreted as focusing on couch components. It also shows a correlation between hand‐pendant failures and MLC failures, which do not appear to have any common properties. The reason for the correlation of failures is that for the linac in question, the hand pendant is used to initiate motions of the MLC as well as other motor actions (gantry, collimator, jaws) through the “move to position” command. If an MLC is not operating correctly, it is often at this stage that the problem presents itself, which can be initiated by the users as a hand‐pendant failure. In some instances, this is an actual hand‐pendant failure, but in other instances, the root cause of the failure is a faulty MLC. Hence, in our processing of the text descriptions of the fault logs, we have seen a correlation between these two component failures.

#### 3.2.2 Increasing pattern

**FIGURE 5:**
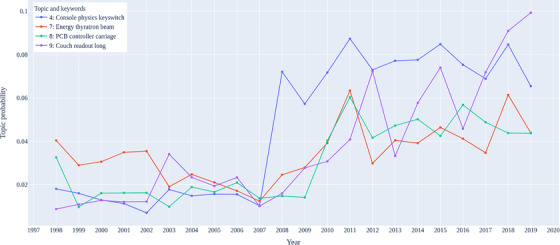
Trends of topics with increasing trend over years

**FIGURE 6:**
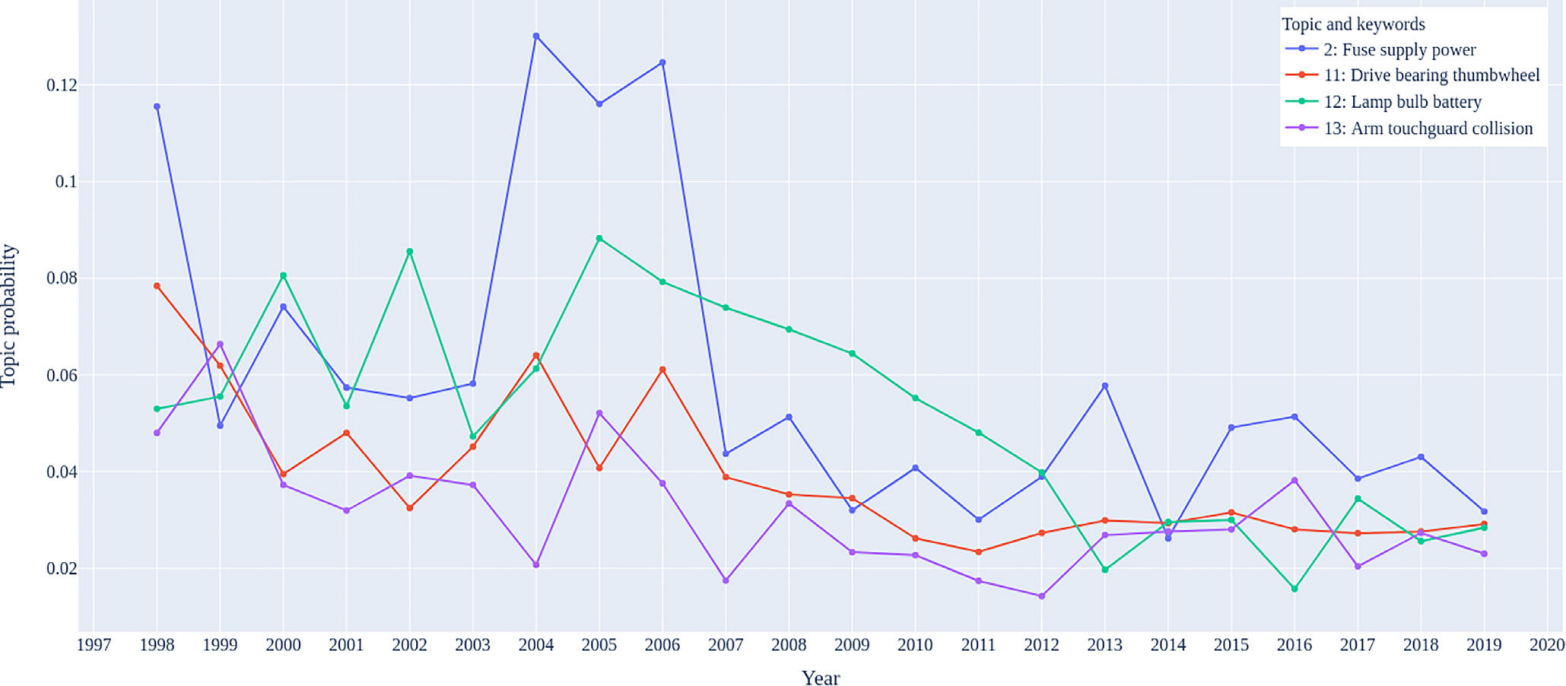
Trends of topics with decreasing trend over years

Except for the periodic pattern, as shown in Figures 5 and 6, some topics exhibited quasi‐monotonic change over the past 20 years. Most noticeable in these figures is the distinction between the trend of failure topics before and after about 2009. As mentioned in Section 2, the cancer clinic underwent linac replacements starting from 2009 to 2011 in which the linacs, as well as linac manufacturers, were changed. This time‐frame corresponds to the trends shown in Figures 5 and 6 which show that several failure topics had significant increases or decreases between 2009 and 2011.

Figure 5 shows the estimated proportion of topics 4, 7, 8, and 9 over time with a one‐year interval. In the review of the graphic trend, a significant increase around 2009 was noticed, consistent with the linacs replacement history. Although the failure modes reflected from these topics belong to several subsystems, they can all be linked to the malfunction of the linac controlling somehow. Specifically, this change happened to the topics and failure modes involved can be explained as below:

1. *Topic 4*: Console keyswitch failure. The linear accelerators in the period 1998–2009 did not have a keyswitch.
2. *Topic 7*: Thyratron failure; De‐quing and Thyratron voltage low. The high‐voltage modulator of the two linac systems is quite different as one system used a magnetron‐driven modulator (period 1998–2009) and the other a klystron‐driven system (2011 onward). It appears from the data that failures of the thyratron with the klystron‐based systems are more frequent.
3. *Topic 8*: Replaced controller printed circuit board (PCB). This component is only used in linacs after replacement, from 2011 onward.
4. *Topic 9*: Mechanical calibrations: couch, gantry, collimator. There is no specific reason why mechanical failures may be more prevalent with one linac manufacturer, however it appears as though the linac manufacturer of the units from 2011 onward is more susceptible to these types of failures.

#### 3.2.3 Decreasing pattern

Figure 6 shows the estimated proportion of topic 2, 11, 12, and 13 over with one‐year interval. Similarly, a distinction was found around 2009, the difference is the decreasing trend was developed. Overall, these topics are related to the power support systems and the lighting system.

1. *Topic 2*: High‐voltage modulator components failure. In particular, magnetron failures and modulator relay failures peaked in the years before replacement of the original units. The fact that the replacement linacs did not use magnetrons caused the drop of this topic proportion.
2. *Topic 11*: Couch failures and hand‐pendant failures. This appears to be more stable on the units from 2011 onward than with the original units.
3. *Topic 12*: Lamps and lighting failures. On both manufacturers, these were switched from incandescent bulbs to light‐emitting diode (LED) over this time in this study, which would result in fewer failures as LED lights have a longer lifetime than incandescent bulbs.
4. *Topic 13*: Orthovoltage unit failure. This unit had more kV and generator faults later in its life span.

## 4 DISCUSSION

This study sets out to apply NLP techniques to analyze narrative maintenance logs of linacs. The most significant finding to emerge from the analysis is that the TM is capable of mining failure modes topics and latent components automatically. Compared to the traditional manual inspection, with the application of the TM method, the analysis efficiency was greatly increased, where the data scale is large and the data are recorded without standardized terminology. Another main finding is that linacs from different manufacturers are prone to different failures. One unanticipated finding was that some failures may exhibit a quasi‐synchronous mode over years. For example, the MLC leaf and mechanical components of the couch demonstrate a synchronous failure occurrence. Technicians accounting for the maintenance work of linacs can have a better understanding of the main linac failures. These results further support the analysis of the cause of downtime from a quantitative view.

Other than in the area of linac malfunction classification, NLP can have many other applications in understanding radiotherapy failures. For instance, the incident reporting databases used by ASTRO's RO‐ILS; NSIR‐RT in Canada; and ROSEIS for ESTRO rely on a pre‐defined taxonomy and classification scheme to classify radiotherapy incidents. Incidents that do not fit into a pre‐defined classification framework can be misclassified, which reduces the effectiveness of the incident reporting system. NLP could be useful in this area if it were applied to the comments in incident reporting systems, where common topics could be identified and used to refine the classification scheme to keep it relevant and accurate. This could help convert the unstructured data in narratives into structured data that can be automatically analyzed and used for system improvements. Such methods are used in other areas of radiotherapy and could be adapted to incident reporting.^21^

Some limitations are also revealed by this article. The dataset used in this article was collected from one cancer center with only five linacs in service. Limited by the number of the maintenance service and linacs, only 4323 entries are analyzed. Theoretically, there is no wide agreement on how much data are needed for TM. Two major factors are the length of words in each entry and the complexity of the whole corpus. More words in each entry and more entries can support analyzing a more complex corpus. Table 4 listed the dataset profile of several TM applications in recently published researches, which indicate that dozens of thousands of entries are recommended for the TM analysis. It should be noted that although we only have 4323 entries with an average length of 25.3 words, a reasonable result was still obtained. Two reasons account for this result: (1) the sentence in maintenance logs is simple, which are (2) the scope of the maintenance log corpus is narrower compared to other researches. For example, the aviation safety report contains the aircraft behavior, weather condition, pilot's actions, etc. However, we believe that a larger dataset can support to analyze failure modes more comprehensive and even extend the analysis to a wider scope. Therefore, future research would be placed on taking more types of available data into account. One promising way to address the data scarcity is to analyze the daily report and communications between technicians. These resources are usually recorded in emails. Another limitation of applying TM is the need for expert knowledge to identify information of interest. There is a need to make the analysis procedure automatic and free of experts involved and thus increase the generalization ability of the TM methods. One possible idea is to construct a standardized terminology dictionary for a specific analysis task. A keyword matching procedure can be employed automatically between the outputs of the TM model and dictionary instead of manually inspection.

**TABLE 4:** Dataset used in recent published applications using topic modeling

Number of entries	Average length of each entry	Number of topics	Research field	Reference
55,612	Unknown	30	Patient online review analysis	[5]
19,543	28.3	10	Product review analysis (wireless mouse)	[14]
30,268	27.6	10	Product review analysis (diffuser)	[14]
25,706	73.5	17	Aviation safety report analysis	[3]

## 5 CONCLUSION

This article applied NLP techniques to medical linac maintenance logs to identify frequent failure modes in daily use. Specifically, the TM method was used to identify failure modes and the temporal analysis method was used to investigate the trends of different failure modes frequency over time. The results demonstrated that TM applied here is able to identify the most frequent failure modes of linacs in a quantitative way instead of relying on the experience of maintenance personnel. In our case, the major failure modes are MLC leaf stuck, power subsystem failure, and gantry position adjustment. The temporal analysis allows analysts to discern whether a failure mode has a periodic, increasing, or decreasing trend over time. It is shown that the frequency of MLC failure and couch failure has a relatively consistent synchronization. Linacs with different structures or from different manufacturers may prone to different failure modes in daily use.

## AUTHOR CONTRIBUTIONS

Hongguang Yun: Conceived the idea of the study, designed and conducted the experiments, performed the analysis, and wrote the paper. Marco Carlone: Collected the data, conceived and validated the analysis, and wrote the paper. Zheng Liu: Conceived the idea of the study, designed the experiments, and proofed the results; All authors contributed to revising the paper.

## CONFLICT OF INTEREST

The authors declare that there is no conflict of interest that could be perceived as prejudicing the impartiality of the research reported

## Data Availability

Research data are not shared.
